# Response to Comment on “Heterogeneity in PD-L1 expression in malignant peritoneal mesothelioma with systemic or intraperitoneal chemotherapy”

**DOI:** 10.1038/s41416-020-01215-7

**Published:** 2020-12-14

**Authors:** Michael G. White, Oliver S. Eng

**Affiliations:** 1 Department of Surgical Oncology, The University of Texas MD Anderson Cancer Center, Houston, TX USA; 2 Department of Surgery, Section of General Surgery and Surgical Oncology, University of Chicago, Chicago, IL USA

**Keywords:** Surgical oncology, Mesothelioma

We thank Dr. Yang and colleagues for their discussion and Letter to the Editor regarding our manuscript “Heterogeneity in PD-L1 expression in malignant peritoneal mesothelioma with systemic or intraperitoneal chemotherapy”.^1^ Excellent points are made that highlight the foundation for future clinical trials studying malignant pleural and peritoneal mesothelioma.

The sarcomatoid subtype is certainly an intriguing and challenging subset in patients with malignant peritoneal mesothelioma. Our omission of this subtype was not intentional, but rather a reflection of patients not having been surgically treated during the study period. As the sarcomatoid subtype is known to be more aggressive and a poor prognostic factor even with cytoreductive surgery, a better understanding of this disease may help potentially improve outcomes.^2^ The T-cell infiltrate referenced is interesting as a marker of potential responsiveness along with a variety of immune cell infiltrates. In future work we hope to better define this as well as other factors of the tumour microenvironment (TME) across mesothelioma histologies. Of particular interest to our group moving forward is the potential role of B-cells and the identification of tertiary lymphoid structures (TLS) within mesothelioma. These markers have been demonstrated to portend improved responsiveness to melanoma, sarcoma, and renal cell carcinoma treated with immunotherapy, although their functional role continues to be elucidated.^3–5^ The effect of the pressure of either immunomodulatory or cytotoxic therapies have on these factors of the TME are especially fascinating as we observe changes in the TME longitudinally.

Yang et al. reference their well-written manuscript describing inactivation of tumour suppressor genes and differences in the TME based on these findings. Moreover, they suggest a potential pathway for selection criteria using their findings to select patients with the highest likelihood to respond to immune checkpoint blockade (ICB). This and other work provide a hopeful future direction of therapy selection using histology augmented by molecular profiling rather than histology alone. We have seen similar benefits to this and other treatment strategies in the targeted utilisation of ICB in more common malignancies, such as melanoma, colorectal cancer and breast cancer.^6–8^ This demonstrates that patient selection is critical to optimise outcomes for those patients who can benefit from ICB therapies while allowing those patients whose tumours are unlikely to respond to explore other treatment avenues. A caveat to this line of discussion, however, is that the above observations were made in pleural rather than peritoneal mesothelioma. The immune microenvironment of the peritoneum remains poorly understood, and the discordance in response rates to ICB between pleural and peritoneal mesothelioma is a reflection of this.^9^ Only recently have we begun to describe the immune cellular profile in peritoneal fluid during cytoreductive surgery and hyperthermic intraperitoneal chemotherapy, and preclinical data with novel therapeutics has yielded immune responses which may reflect efficacy.^10,11^

Ongoing discussions and collaborations are crucial to the optimisation of the care of patients with rare diseases such as mesothelioma. Given the low incidence, changes in treatment that adapt to the dynamic landscape of checkpoint inhibition and targeted therapies often require multi-institutional efforts, and in many instances, across nations.

## Data Availability

Not applicable.
